# Editorial: Reliability and Reproducibility in Functional Connectomics

**DOI:** 10.3389/fnins.2019.00117

**Published:** 2019-02-20

**Authors:** Xi-Nian Zuo, Bharat B. Biswal, Russell A. Poldrack

**Affiliations:** ^1^Key Laboratory of Brain and Education, Nanning Normal University, Nanning, China; ^2^Department of Psychology, University of Chinese Academy of Science, Beijing, China; ^3^CAS Key Laboratory of Behavioral Sciences, Institute of Psychology, Beijing, China; ^4^Magnetic Resonance Imaging Research Center, CAS Institute of Psychology, Beijing, China; ^5^Research Center for Lifespan Development of Mind and Brain, CAS Institute of Psychology, Beijing, China; ^6^Institute for Brain Research and Rehabilitation, South China Normal University, Guangzhou, China; ^7^The Clinical Hospital of Chengdu Brain Science Institute, MOE Key Lab for Neuroinformation, University of Electronic Science and Technology of China, Chengdu, China; ^8^Department of Biomedical Engineering, New Jersey Institute of Technology, Newark, NJ, United States; ^9^Department of Psychology, Stanford University, Stanford, CA, United States

**Keywords:** test-retest reliability, functional connectomics, open science, dynamic brain theory, big data

Research on functional connectomics of the human brain is exploding (Kelly et al., 2012; Smith et al., 2013), especially for clinical and neurodevelopmental as well as aging studies. However, advances in the reliability and validity of functional connectomics have so far lagged the application of these methods in practice (Zuo and Xing, 2014). In statistical theory, reliability serves as an upper limit of validity and is measurable in practice while validity is more difficult to measure directly (e.g., specific trait and disease) thus often approximated by predictive validity (Kraemer, 2014). Therefore, high reliability is a required standard for both research and clinical use. Of note, excellent reliability (>0.8) serves the clinical standard on measurement scales (Streiner et al., 2015). This reflects clinical call of tools with high inter-individual differences (*easily differentiating individuals*) and low intra-individual differences (*high individual stability*) (Fleiss et al., 2003; Zuo and Xing, 2014). This has been recently demonstrated in the anatomy of reliability (Xing and Zuo, 2018). In reliability studies, statistical quantification of reliability is often implemented with intracclass correlation (ICC) regarding its well-developed theory in the field of probability and statistics while the types of ICC are determined by the repeated-measure experimental design (Shrout and Fleiss, 1979; Koo and Li, 2016). Failure of reliability can be an important cause of small statistical power (Button et al., 2013), low reproducibility (Poldrack et al., 2017), puzzlingly high correlations (Vul et al., 2009), and overwhelming need of big data or large sample sizes (Streiner et al., 2015; Hedge et al., 2018). In the field of human brain mapping with magnetic resonance imaging (MRI), structural MRI has clinically-acceptable reliability of mapping brain morphology (Madan and Kensinger, 2017) while most functional MRI measures are challenged by the clinical standard on the reliability (Bennett and Miller, 2010; Zuo and Xing, 2014). This research topic takes action on further steps of improving the reliability of fMRI-based connectomics by publishing 12 papers across experimental design, computational algorithm, and brain dynamics theory.

Given the sensitivity of resting-state fMRI (rfMRI) connectivity measurements to physiological variables, the development of improved strategies for correction of physiological artifacts is imperative. Golestani et al. demonstrated significant improvements of reproducibility of common rfMRI metrics by the low-frequency physiological correction with end-tidal CO_2_. Related to human arousal, as demonstrated in Wang et al., test-retest reliability of human functional connectomics can be significantly improved by removing the impact of sleep using measures of heart rate variability derived from simultaneous electrocardiogram recording. These findings highlight the need of recordings of physiological variables for reproducible functional connectomics. In addition, the use of eyes-open versus eyes-closed resting is an important aspect of rfMRI experimental design and has been of great research interest due to its relationships with visual function (Yang et al., 2007) and arousal (Yan et al., 2009; Tagliazucchi and Laufs, 2014). The study by Yuan et al. provides a novel multivariate method to examine the amplitude differences of brain oscillations between eyes open and eyes close conditions during resting state as well as their scanner-related reliability. Head motion during scanning is another potential source of variability and has been relatively well investigated regarding its impacts on reliability of rfMRI derivatives by using various preprocessing strategies (Yan et al., 2013; Ciric et al., 2017; Parkes et al., 2018). Furthermore, how these variables are modeled and the order in the preprocessing pipelines they are modeled can have significant impacts on results (Chen et al., 2017; Lindquist et al., 2019). These advances have implications on the way of further optimizing the reliability observed (Golestani et al.; Wang et al.).

Many computational algorithms exist for characterizing features of the organization in the functional connectomes across different spatial and temporal scales (Zuo and Xing, 2014). Reliability can guide both methodological choices between these algorithms as well as the validation of new algorithms. Common algorithms have been recently given a state of art review in terms of their test-retest reliability (Zuo and Xing, 2014), indicating that network metrics derived from graph theory applied to rfMRI signal are less reliable (Zuo et al., 2012) than usually required while both local functional homogeneity measure (Zuo et al., 2013) and global network measure with dual regression of independent component analysis (drICA) (Zuo et al., 2010a) almost reach the clinical standard of reliability. This topic offers five studies to illustrate more sophisticated developments of reliability of these algorithms. This topic proposed a novel algorithm for network generation at individual level, using topological filtering based on orthogonal minimal spanning trees to show both functional and structural networks with highly reliable graph theoretical measures using magnetoencephalography (Dimitriadis et al.) and diffusion MRI (Dimitriadis et al.). Reliability evaluations are comprehensively investigated for group information guided ICA, independent vector analysis (IVA) (Du et al.). and other high-order functional connectivity (Zhang et al.). The single-subject spatially-constrained ICA performs favorably compared to IVA (Du et al.) and improves detection of clinical differences compared to drICA (Salman et al., 2018). Additionally, Di and Biswal warned the field by demonstrating the poor reliability of using psychophysiological interaction analyses in the context of inter-individual correlation or group comparisons.

As commented by Sato et al., open science with sharing of large datasets has paved the way for delineating the fingerprints of human brain function. This is reflected by the fact that most studies in the topic employed the data from Consortium for Reliability and Reproducibility (Zuo et al., 2014), representing a means of accelerating science by facilitating collaboration, transparency, and reproducibility (Milham et al., 2018). To address the reproducibility issue in the field of human brain mapping, the Organization for Human Brain Mapping (OHBM) have created a Committee on Best Practices in Data Analysis and Sharing (COBIDAS) and published its report (Nichols et al., 2017). Beyond the advances, two studies also raised challenges of big-data applications to clinical population, particularly in understanding the high heterogeneity of spontaneous brain activity in ADHD and autism (Wang et al.; Syed et al.). As noted in Button et al. (2013), large samples may produce statistically significant results even for extremely small effects which have little add to diagnostic or clinical utility. These observable but small effects are likely caused by weighing the low measurement reliability with the true effect (Streiner et al., 2015), which could be moderate to large. It is thus very fundamental to estimate effect size in neuroimaging and its relationship with statistical power although most existing studies have not factored the reliability in doing so (Reddan et al., 2017; Geuter et al., 2018). This is particularly valuable for some widely used but less reliable measures (e.g., seed-based functional connectivity) (Shou et al., 2013; Zuo and Xing, 2014; Siegel et al., 2017) to be improved with acceptable reliability ahead of its clinical use (Fox, 2018). Meanwhile, data harmonization techniques such as ComBat (Yu et al., 2018) should be developed to reduce inter-scan or inter-site differences in multi-center big-data studies. One possibility of filling these gaps between empirical computation and clinical application is theoretical development of brain dynamics (Woo et al., 2017). The work by Tomasi et al. demonstrated a power law of the brain network dynamics, which has been framed into a theory of neural oscillations (Buzsáki and Draguhn, 2004). Combination of theory and data via structure-function fusion (Zuo et al., 2010b; Jiang and Zuo, 2016) will remove the reliability barriers of developing clinically useful human brain mapping, which is the final call of the current research topic.

## Author Contributions

X-NZ drafted the editorial and worked on the revisions with BB and RP.

### Conflict of Interest Statement

The authors declare that the research was conducted in the absence of any commercial or financial relationships that could be construed as a potential conflict of interest.

## References

[B1] Bennett C. M. Miller M. B. (2010). How reliable are the results from functional magnetic resonance imaging? Ann. N.Y. Acad. Sci. 1191, 133–155. 10.1111/j.1749-6632.2010.05446.x 20392279

[B2] Button K. S. Ioannidis J. P. Mokrysz C. Nosek B. A. Flint J. Robinson E. S.. (2013). Power failure: why small sample size undermines the reliability of neuroscience. Nat. Rev. Neurosci. 14, 365–376. 10.1038/nrn3475 23571845

[B3] Buzsáki G. Draguhn A. (2004). Neuronal oscillations in cortical networks. Science 304, 1926–1929. 10.1126/science.1099745 15218136

[B4] Chen J. E. Jahanian H. Glover G. H. (2017). Nuisance regression of high-frequency functional magnetic resonance imaging data: denoising can be noisy. Brain Connect. 7, 13–24. 10.1089/brain.2016.0441 27875902 PMC5312601

[B5] Ciric R. Wolf D. H. Power J. D. Roalf D. R. Baum G. L. Ruparel K.. (2017). Benchmarking of participant-level confound regression strategies for the control of motion artifact in studies of functional connectivity. Neuroimage 154, 174–187. 10.1016/j.neuroimage.2017.03.020 28302591 PMC5483393

[B6] Fleiss J. L. Levin B. Paik M. C. (2003). Statistical Methods for Rates and Proportions, 3rd edn. Wiley Series in Probability and Statistics. Hoboken, NJ: John Wiley & Sons.

[B7] Fox M. D. (2018). Mapping symptoms to brain networks with the human connectome. New Engl. J. Med. 379, 2237–2245. 10.1056/NEJMra1706158 30575457

[B8] Geuter S. Qi G. Welsh R. C. Wager T. D. Lindquist M. A. (2018). Effect size and power in fMRI group analysis. bioRxiv [Preprint]. bioRxiv:295048. 10.1101/295048

[B9] Hedge C. Powell G. Sumner P. (2018). The reliability paradox: why robust cognitive tasks do not produce reliable individual differences. Behav. Res. Methods 50, 1166–1186. 10.3758/s13428-017-0935-1 28726177 PMC5990556

[B10] Jiang L. Zuo X. N. (2016). Regional homogeneity: a multimodal, multiscale neuroimaging marker of the human connectome. Neuroscientist 22, 486–505. 10.1177/1073858415595004 26170004 PMC5021216

[B11] Kelly C. Biswal B. B. Craddock R. C. Castellanos F. X. Milham M. P. (2012). Characterizing variation in the functional connectome: promise and pitfalls. Trends Cogn. Sci. 16, 181–188. 10.1016/j.tics.2012.02.001 22341211 PMC3882689

[B12] Koo T. K. Li M. Y. (2016). A guideline of selecting and reporting intraclass correlation coefficients for reliability research. J. Chiropr. Med. 15, 155–163. 10.1016/j.jcm.2016.02.012 27330520 PMC4913118

[B13] Kraemer H. C. (2014). The reliability of clinical diagnoses: state of the art. Annu. Rev. Clin. Psychol. 10, 111–130. 10.1146/annurev-clinpsy-032813-153739 24387235

[B14] Lindquist M. A. Geuter S. Wager T. D. Caffo B. S. (2019). Modular preprocessing pipelines can reintroduce artifacts into fMRI data. Human Brain Mapp. 10.1002/hbm.24528 [Epub ahead of print]. 30666750 PMC6865661

[B15] Madan C. R. Kensinger E. A. (2017). Test–retest reliability of brain morphology estimates. Brain Inform. 4, 107–121. 10.1007/s40708-016-0060-4 28054317 PMC5413592

[B16] Milham M. P. Craddock R. C. Son J. J. Fleischmann M. Clucas J. Xu H.. (2018). Assessment of the impact of shared brain imaging data on the scientific literature. Nat. Commun. 9:2818. 10.1038/s41467-018-04976-1 30026557 PMC6053414

[B17] Nichols T. E. Das S. Eickhoff S. B. Evans A. C. Glatard T. Hanke M.. (2017). Best practices in data analysis and sharing in neuroimaging using MRI. Nat. Neurosci. 20, 299–303. 10.1038/nn.4500 28230846 PMC5685169

[B18] Parkes L. Fulcher B. Yücel M. Fornito A. (2018). Benchmarking of participant-level confound regression strategies for the control of motion artifact in studies of functional connectivity. Neuroimage 171, 415–436. 10.1016/j.neuroimage.2017.12.073 28302591 PMC5483393

[B19] Poldrack R. A. Baker C. I. Durnez J. Gorgolewski K. J. Matthews P. M. Munafò M. R.. (2017). Scanning the horizon: towards transparent and reproducible neuroimaging research. Nat. Rev. Neurosci. 18, 115–126. 10.1038/nrn.2016.167 28053326 PMC6910649

[B20] Reddan M. C. Lindquist M. A. Wager T. D. (2017). Effect size estimation in neuroimaging. JAMA Psychiatry 74, 207–208. 10.1001/jamapsychiatry.2016.3356 28099973

[B21] Salman M. S. Du Y. Lin D. Fu Z. Damaraju E. Sui J. (2018). Group ICA for identifying biomarkers in schizophrenia: ‘adaptive’ networks via spatially constrained ICA show more sensitivity to group differences than spatio-temporal regression. bioRxiv [Preprint]. bioRxiv:429837. 10.1101/429837 PMC6438914 30921608

[B22] Shou H. Eloyan A. Lee S. Zipunnikov V. Crainiceanu A. N. Nebel N. B.. (2013). Quantifying the reliability of image replication studies: the image intraclass correlation coefficient (I2C2). Cogn. Affect. Behav. Neurosci. 13, 714–724. 10.3758/s13415-013-0196-0 24022791 PMC3869880

[B23] Shrout P. E. Fleiss J. L. (1979). Intraclass correlations: uses in assessing rater reliability. Psychol. Bull. 86, 420–428. 10.1037/0033-2909.86.2.420 18839484

[B24] Siegel J. S. Mitra A. Laumann T. O. Seitzman B. A. Raichle M. Corbetta M.. (2017). Data quality influences observed links between functional connectivity and behavior. Cereb. Cortex 27, 4492–4502. 10.1093/cercor/bhw253 27550863 PMC6410500

[B25] Smith S. M. Vidaurre D. Beckmann C. F. Glasser M. F. Jenkinson M. Miller K. L.. (2013). Functional connectomics from resting-state fMRI. Trends Cogn. Sci. 17, 666–682. 10.1016/j.tics.2013.09.016 24238796 PMC4004765

[B26] Streiner D. L. Norman G. R. Cairney J. (2015). Health Measurement Scales: A Practical Guide to Their Development and Use, 5th Edn. New York, NY: Oxford University Press.

[B27] Tagliazucchi E. Laufs H. (2014). Decoding wakefulness levels from typical fMRI resting-state data reveals reliable drifts between wakefulness and sleep. Neuron 82, 695–708. 10.1016/j.neuron.2014.03.020 24811386

[B28] Vul E. Harris C. Winkielman P. Pashler H. (2009). Puzzlingly high correlations in fMRI studies of emotion, personality, and social cognition. Perspect. Psychol. Sci. 4, 274–290. 10.1111/j.1745-6924.2009.01125.x 26158964

[B29] Woo C. W. Chang L. J. Lindquist M. A. Wager T. D. (2017). Building better biomarkers: brain models in translational neuroimaging. Nat. Neurosci. 20, 365–377. 10.1038/nn.4478 28230847 PMC5988350

[B30] Xing X. X. Zuo X. N. (2018). The anatomy of reliability: a must read for future human brain mapping. Sci. Bull. 63, 1606–1607. 10.1016/j.scib.2018.12.010 36658850

[B31] Yan C. Liu D. He Y. Zou Q. Zhu C. Zuo X.. (2009). Spontaneous brain activity in the default mode network is sensitive to different resting-state conditions with limited cognitive load. PLoS ONE 4:e5743. 10.1371/journal.pone.0005743 19492040 PMC2683943

[B32] Yan C. G. Cheung B. Kelly C. Colcombe S. Craddock R. C. Martino A. D.. (2013). A comprehensive assessment of regional variation in the impact of head micromovements on functional connectomics. Neuroimage 76, 183–201. 10.1016/j.neuroimage.2013.03.004 23499792 PMC3896129

[B33] Yang H. Long X. Y. Yang Y. Yan H. Zhu C. Z. Zhou X. P.. (2007). Amplitude of low frequency fluctuation within visual areas revealed by resting-state functional MRI. Neuroimage 36, 144–152. 10.1016/j.neuroimage.2007.01.054 17434757

[B34] Yu M. Linn K. A. Cook P. A. Phillips M. L. McInnis M. Fava M.. (2018). Statistical harmonization corrects site effects in functional connectivity measurements from multi-site fMRI data. Hum. Brain Mapp. 39, 4213–4227. 10.1002/hbm.24241 29962049 PMC6179920

[B35] Zuo X. N. Anderson J. S. Bellec P. Birn R. M. Biswal B. B. Blautzik J.. (2014). An open science resource for establishing reliability and reproducibility in functional connectomics. Sci. Data 1:140049. 10.1038/sdata.2014.49 25977800 PMC4421932

[B36] Zuo X. N. Ehmke R. Mennes M. Imperati D. Castellanos F. X. Sporns O.. (2012). Network centrality in the human functional connectome. Cereb. Cortex 22, 1862–1875. 10.1093/cercor/bhr269 21968567

[B37] Zuo X. N. Kelly C. Adelstein J. S. Klein D. F. Castellanos F. X. Milham M. P. (2010a). Reliable intrinsic connectivity networks: test-retest evaluation using ICA and dual regression approach. Neuroimage 49, 2163–2177. 10.1016/j.neuroimage.2009.10.080 19896537 PMC2877508

[B38] Zuo X. N. Kelly C. Martino A. D. Mennes M. Margulies D. S. Bangaru S.. (2010b). Growing together and growing apart: regional and sex differences in the lifespan developmental trajectories of functional homotopy. J. Neurosci. 30, 15034–15043. 10.1523/JNEUROSCI.2612-10.2010 21068309 PMC2997358

[B39] Zuo X. N. and Xing, X. X. (2014). Test-retest reliabilities of resting-state FMRI measurements in human brain functional connectomics: a systems neuroscience perspective. Neurosci. Biobehav. Rev. 45, 100–118. 10.1016/j.neubiorev.2014.05.009 24875392

[B40] Zuo X. N. Xu T. Jiang L. Yang Z. Cao X. Y. He Y.. (2013). Toward reliable characterization of functional homogeneity in the human brain: preprocessing, scan duration, imaging resolution and computational space. Neuroimage 65, 374–386. 10.1016/j.neuroimage.2012.10.017 23085497 PMC3609711

