# Corrigendum: Editorial: Reliability and Reproducibility in Functional Connectomics

**DOI:** 10.3389/fnins.2019.00374

**Published:** 2019-04-16

**Authors:** Xi-Nian Zuo, Bharat B. Biswal, Russell A. Poldrack

**Affiliations:** ^1^Key Laboratory of Brain and Education, Nanning Normal University, Nanning, China; ^2^Department of Psychology, University of Chinese Academy of Science, Beijing, China; ^3^CAS Key Laboratory of Behavioral Sciences, Institute of Psychology, Beijing, China; ^4^Magnetic Resonance Imaging Research Center, CAS Institute of Psychology, Beijing, China; ^5^Research Center for Lifespan Development of Mind and Brain, CAS Institute of Psychology, Beijing, China; ^6^Institute for Brain Research and Rehabilitation, South China Normal University, Guangzhou, China; ^7^The Clinical Hospital of Chengdu Brain Science Institute, MOE Key Lab for Neuroinformation, University of Electronic Science and Technology of China, Chengdu, China; ^8^Department of Biomedical Engineering, New Jersey Institute of Technology, Newark, NJ, United States; ^9^Department of Psychology, Stanford University, Stanford, CA, United States

**Keywords:** test-retest reliability, functional connectomics, open science, dynamic brain theory, big data

In the original article, we neglected to include the funder “Guangxi BaGui Scholarship, 201621” to X-NZ.

In the published article, there was an error regarding the affiliation for Bharat Biswal. As well as having affiliation 8, he should also have “The Clinical Hospital of Chengdu Brain Science Institute, MOE Key Lab for Neuroinformation, University of Electronic Science and Technology of China, Chengdu, China.”

The authors apologize for this error and state that this does not change the scientific conclusions of the article in any way. The original article has been updated.

